# Improvement of physical properties of calcium phosphate cement by elastin-like polypeptide supplementation

**DOI:** 10.1038/s41598-018-23577-y

**Published:** 2018-03-26

**Authors:** Ji-Hyun Jang, Sumi Shin, Hyun-Jung Kim, Jinyoung Jeong, Hyo-Eon Jin, Malav S. Desai, Seung-Wuk Lee, Sun-Young Kim

**Affiliations:** 1 Department of Conservative Dentistry, School of Dentistry, Kyung Hee University, Seoul, Korea; 2 Department of Conservative Dentistry, Graduate School, Kyung Hee University, Seoul, Korea; 3 Hazards Monitoring BNT Research Center, Korea Research Institute of Bioscience and Biotechnology, KRIBB School, University of Science and Technology, Daejon, Korea; 4 College of Pharmacy, Ajou University, Suwon, Korea; 5 Department of Bioengineering, University of California, Berkeley, USA; 6 Department of Conservative Dentistry and Dental Research Institute, School of Dentistry, Seoul National University, Seoul, Korea

## Abstract

Calcium phosphate cements (CPCs) are synthetic bioactive cements widely used as hard tissue substitutes. Critical limitations of use include their poor mechanical properties and poor anti-washout behaviour. To address those limitations, we combined CPC with genetically engineered elastin-like polypeptides (ELPs). We investigated the effect of the ELPs on the physical properties and biocompatibility of CPC by testing ELP/CPC composites with various liquid/powder ratios. Our results show that the addition of ELPs improved the mechanical properties of the CPC, including the microhardness, compressive strength, and washout resistance. The biocompatibility of ELP/CPC composites was also comparable to that of the CPC alone. However, supplementing CPC with ELPs functionalized with octaglutamate as a hydroxyapatite binding peptide increased the setting time of the cement. With further design and modification of our biomolecules and composites, our research will lead to products with diverse applications in biology and medicine.

## Introduction

Calcium phosphate cements (CPCs), which are usually composed of various calcium phosphate powder mixtures and a liquid solution^1^, have been widely used as synthetic bioactive cements. The setting reaction is initiated by hydrolysis between the components, followed by dissolution and reprecipitation^2^. Due to their compositional similarities to hydroxyapatite (HA), CPCs are valued as self-setting, bioactive, osteoconductive, osteotransductive, mouldable or injectable materials. These characteristics make CPCs promising materials for hard tissue engineering such as bone repair, replacement, and regeneration^3,4^. Another beneficial feature of CPCs is their microporous structure, which makes them useful drug delivery vehicle for antibiotics, anti-inflammatory drugs, and antitumor drugs^1,5^. They are also used to develop dental materials for the augmentation or repair of alveolar bone, tooth replacement, root surface desensitization, and root canal fillings^4^.

An ideal material for hard tissue engineering should have the following characteristics: good mechanical strength to withstand loads, appropriate setting time, and favourable anti-washout capability to maintain stability in biological fluids. Despite their biocompatibility and easy applicability, the poor mechanical properties of CPCs limit their application at load-bearing sites^3,6^. Moreover, their low cohesion means that CPCs are easily disrupted on premature contact with biological fluids or blood, leading to undesired washout^7^.

To overcome these limitations, several formulations of CPCs with the addition of various chemicals have been suggested^6^. The addition of citric acid enhances the mechanical strength of CPCs^8,9^, and other studies have demonstrated that the use of chitosan and glucose with citric acid further improve the mechanical properties of CPC cements^10,11^. Polysaccharides such as chitosan and sodium alginate have also been shown to enhance the washout resistance of CPCs. However, these two biopolymers reduce the mechanical strength of CPCs by inhibiting the formation of HA and disturbing the setting process^7,12,13^. Although numerous supplements such as gelatin, albumin, NaCl particles, and glass fibres have been incorporated into CPCs to improve their physical properties, none have completely fulfilled clinical demands^6^.

Protein-based polypeptides (PBPs), biomimetic polymers developed using genetic engineering technology, were recently incorporated into CPCs to enhance the mechanical properties. Some advantages of PBPs include the precise sequence design and chemical composition, monodispersed polymer chains, and access to a vast library of natural and synthetic sequences with unique structural and functional properties^14^. PBPs of particular interest include the elastin-like polypeptides (ELPs), which have a repetitive sequence derived from the mammalian protein elastin. ELPs are composed of repeats of the pentapeptide Val-Pro-Gly-Xaa-Gly; the guest residue Xaa can be any amino acid except Pro^15^. Some important characteristics of ELPs include their outstanding biocompatibility, non-immunogenic properties, and non-toxic and controllable degradation^16,17^. The primary property that makes ELPs unique is their thermoresponsive behaviour, as they can reversibly convert between soluble and insoluble forms around the transition temperature through a phenomenon known as inverse temperature transition. The transition temperature of an ELP can be controlled by the sequence and size of the PBP and by environmental conditions^18,19^. Due to their phase transition and injectability under mild conditions, ELPs have been widely used as promising biomacromolecules for biological tissue engineering and drug delivery vehicles^20^. Wang *et al*. endeavoured to overcome the limitations of CPCs by incorporating genetically engineered ELPs with various target interactions^21^. The novel ELP-based organic/inorganic composites showed improved mechanical properties. The work demonstrated that the augmentation of bioactive cement with ELPs has promising potential applications in development of biomaterials.

Previous research^21^ demonstrated the basic characterization of novel ELP/CPC composites. This study further investigated the effects of ELP supplementation on the physical properties and biocompatibility of CPCs. We evaluated the physical and biological properties of various liquid/powder (L/P) ratios of ELP/CPC combinations to determine a clinically relevant composition as an alternative material for hard tissue engineering.

## Results

### Physical Properties of ELP/CPC Composites

**Figure 1:**
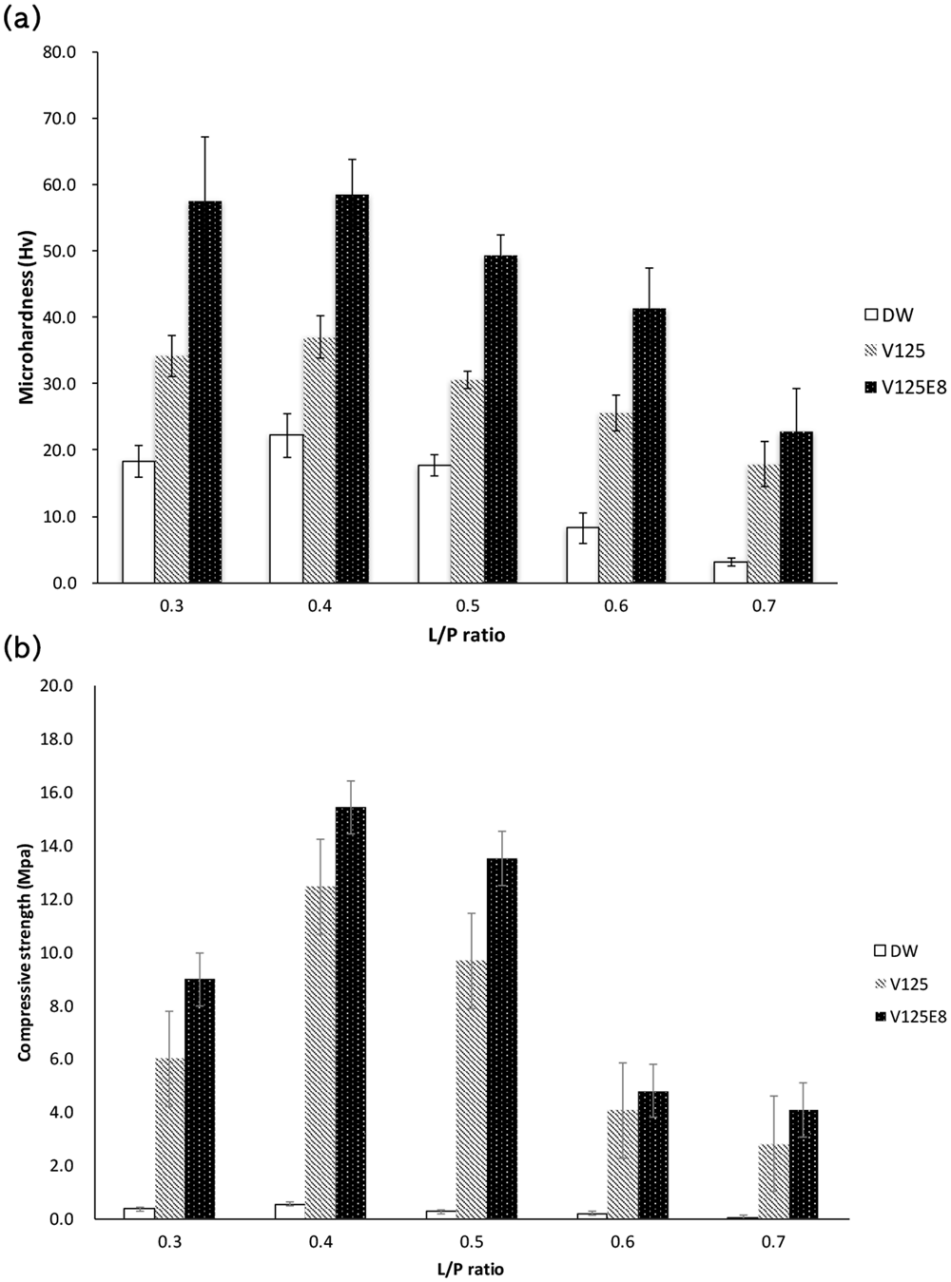
Microhardness (**a**) and compressive strength (**b**) of CPC composites. V125-E8 had the highest values at all L/P ratios; DW had the lowest. V125-E8 had a microhardness 2- to 7-fold higher and a compressive strength >10-fold higher than DW. The microhardness and compressive strength tended to decrease as the L/P ratio increased. However, the highest microhardness and compressive strength were observed at an L/P ratio of 0.4.

First, we compared the microhardness and compressive strength of CPC composites supplemented with two different ELPs (V125 and V125E8) and an ELP-free control (distilled water, DW) at various L/P ratios between 0.3 and 0.7. Figure 1 shows the overall results of the microhardness and compressive strength tests. The incorporation of ELPs significantly increased the microhardness and compressive strength of the CPC regardless of the L/P ratio. The overall tendency showed increased microhardness as follows: V125E8 > V125 > DW at each L/P ratio tested (*p* < 0.05). The V125E8 group had microhardness values 2- to 7-fold higher than the DW group according to the L/P ratio (*p* < 0.05). The V125E8 group also had the highest compressive strength and the DW group had the lowest at all L/P ratios, similar to the trend observed in the microhardness tests. The difference between the V125E8 and DW groups was at least 10-fold greater at L/P ratios of 0.5 and 0.7 (*p* < 0.05). The microhardness and compressive strength generally decreased as the L/P ratio increased for all CPC groups. However, the highest microhardness and compressive strength were observed at an L/P ratio of 0.4.

**Figure 2:**
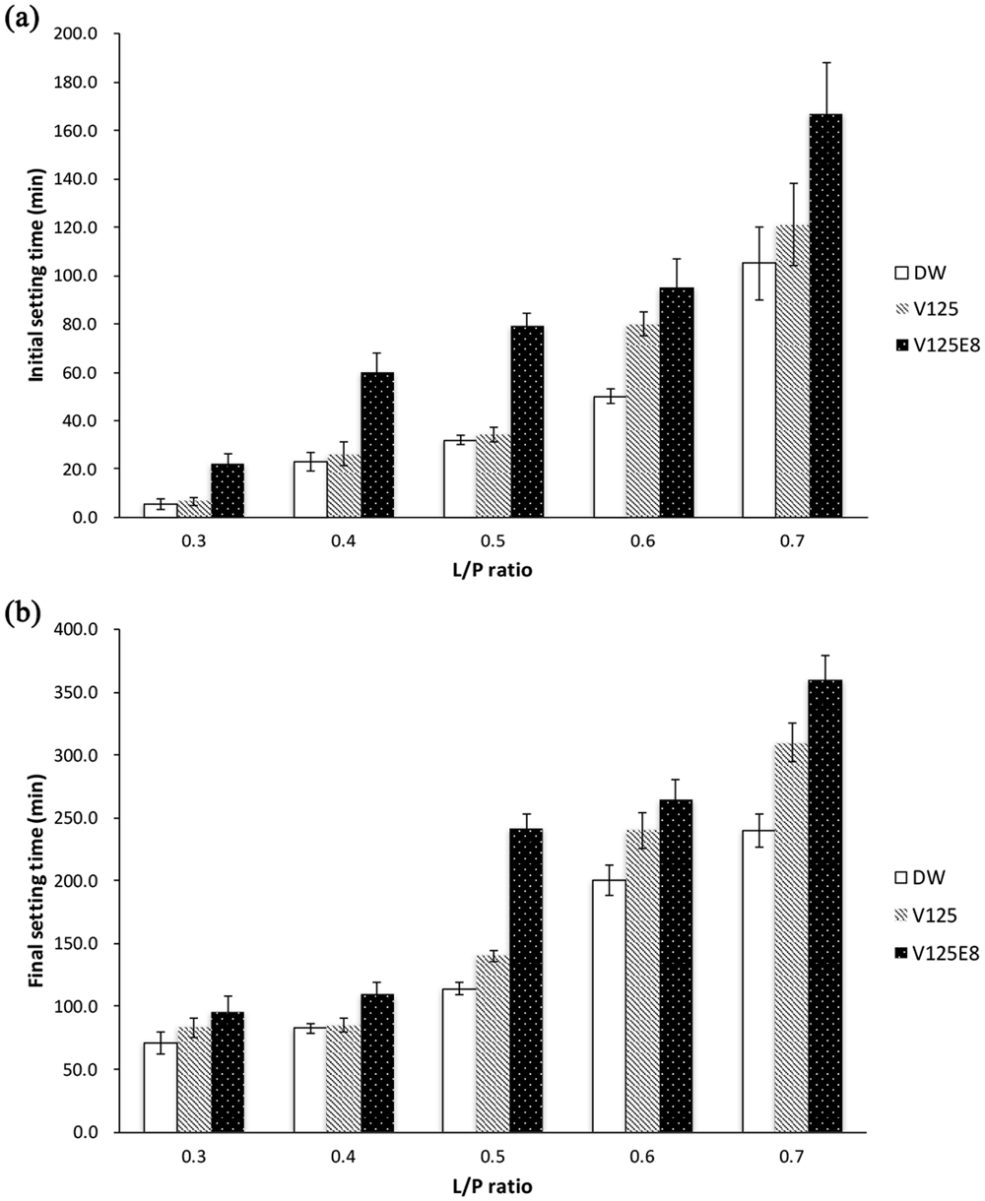
Initial setting time (**a**) and final setting time (**b**). The addition of ELPs increased the initial and final setting times of the CPCs, and V125E8 had a stronger effect than V125. The setting time also increased as the L/P ratio increased.

We also investigated the effects of ELP supplementation on the setting time. It is critical to develop the bioactive cement with the clinically appropriate setting time to decrease the possibility of damage from physical impacts before the cement can set. The effect of the ELP supplement on the initial and final setting times is shown in Fig. 2. The initial and final setting times of the CPC significantly increased with V125E8 supplementation at all L/P ratios compared to the unsupplemented control (*p* < 0.05). V125 supplementation also resulted in significantly increased initial and final setting times at higher L/P ratios of 0.5 to 0.7 (*p* < 0.05). The longest setting time was observed in V125E8 specimens at all L/P ratios tested. The initial and final setting times also increased as the L/P ratio increased for all three types of CPC tested.

**Figure 3:**
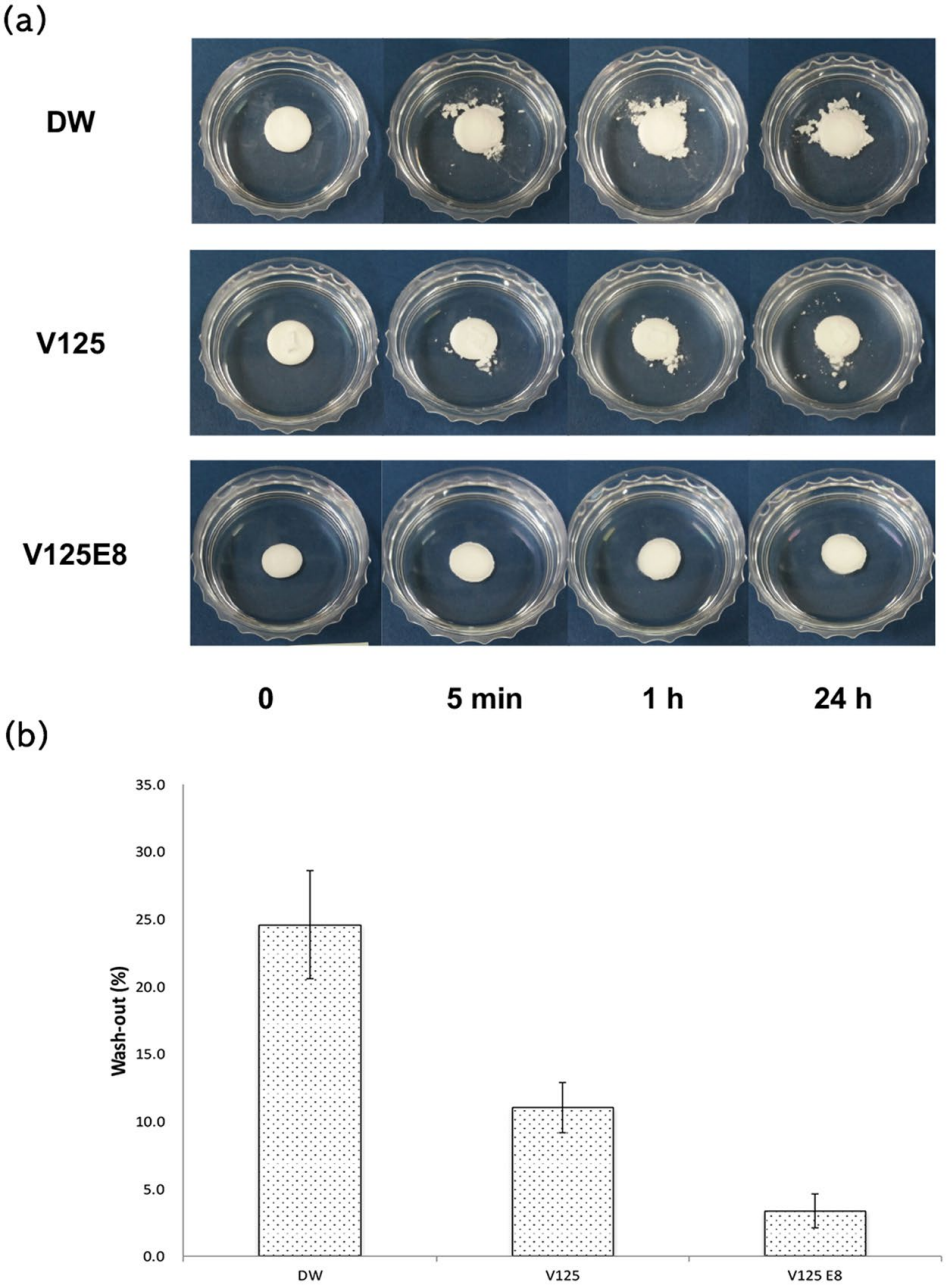
Washout resistance. Appearance (**a**) and the relative amount (**b**) of washed out cement. ELP/CPC composites showed greater washout resistance compared to DW. V125E8 samples tended to maintain their initial shapes; there was no obvious degradation during observation.

Despite the increased setting time due to ELP supplementation, the washout resistance tests of the ELP/CPC composites showed desirable results. The washout resistance tests characterized the dimensional stability of the CPCs by immersion in 4-(2-hydroxyethyl)-1-piperazineethanesulfonic acid (HEPES) solution, which is widely used to maintain physiologic pH. As shown in Fig. 3, DW specimens began to disintegrate after 5 min, and the extent of degradation increased with longer immersion times. The amount of washed out cement after 24 h in the DW group was 24.6 ± 4.0% of the starting material. However, the ELP-supplemented groups demonstrated improved anti-washout characteristics. In particular, V125E8 specimens tended to maintain their initial shapes and no obvious degradation was observed (Fig. 3a). The amount of washed out cement in the V125 and V125E8 groups was 11 ± 1.9% and 3.4 ± 1.3%, respectively. The V125E8 group showed very low washout; it was approximately an order of magnitude lower than that observed in the control group, and one-fourth lower than the V125 group.

**Figure 4:**
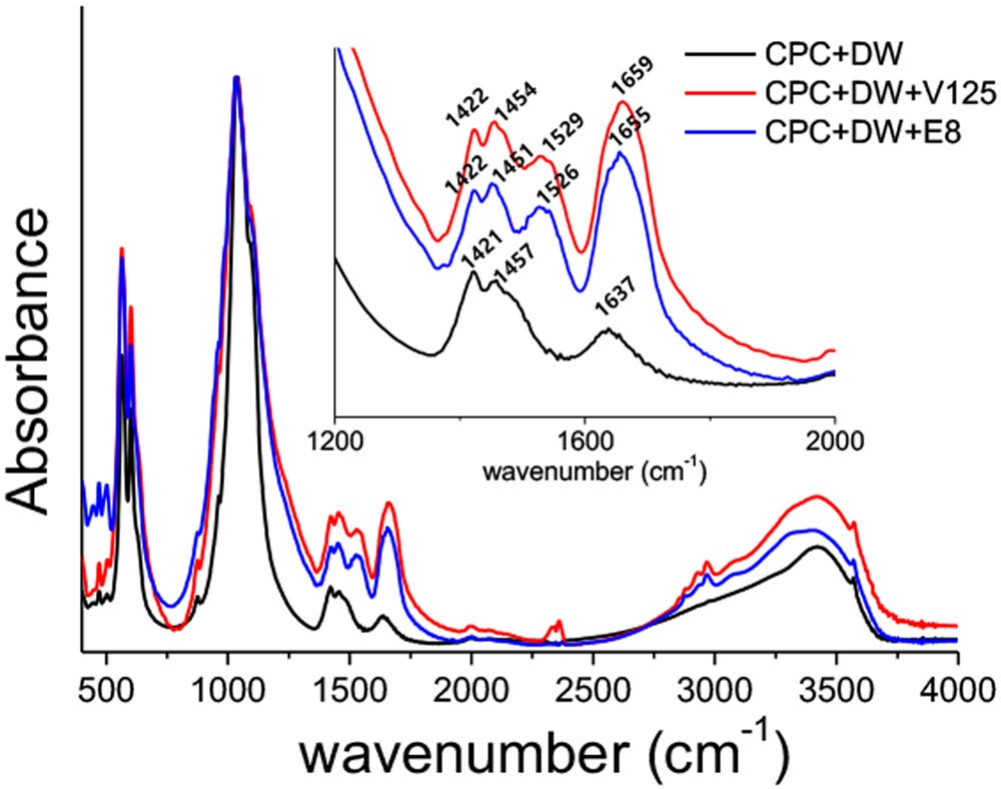
FT-IR analysis. The IR spectra of V125 and V125-E8 showed peaks characteristic of polypeptides, including amide I (1656 cm^−1^) and amide II (1525 cm^−1^), asymmetric sp3 CH2 (2965 cm^−1^), symmetric sp3 CH2 (2875 cm^−1^), and OH (3307 cm^−1^). The peaks around 2300 cm^−1^ in CPC with V125 indicates CO_2_ or H_2_O in the air, which was not removed by background compensation process.

We examined the structure of the experimental ELP/CPC composites via FT-IR spectroscopy and scanning electron microscopy (SEM) to characterize the chemical structural and surface microstructure, respectively. Figure 4 presents the Fourier transform infrared (FT-IR) spectroscopy spectra of the experimental groups. The peaks of dicalcium phosphate anhydrous (DCPA; CaHPO_4_ Ca/P = 1) and tetracalcium phosphate (TTCP; Ca_4_(PO_4_)_2_O, Ca/P = 2), the constituents of CPC powder, were commonly observed in all experimental groups. The characteristic peaks of DCPA were visible at 583 cm^−1^ (PO_4_^3−^), 889 cm^−1^ (HPO_4_^2−^), 993 cm^−1^ (P-O stretching), and 1069 cm^−1^ (P-O stretching). The characteristic peaks of TTCP were visible at 567 cm^−1^ (ν4 PO_4_^3−^), 938 cm^−1^ (ν1 PO_4_^3−^), and 1017 cm^−1^ (ν3 PO_4_^3−^). The IR spectra of the V125 and V125E8 groups showed the characteristic peaks of polypeptides, including amide I (1656 cm^−1^) and amide II (1525 cm^−1^), asymmetric sp3 CH2 (2965 cm^−1^), symmetric sp3 CH_2_ (2875 cm^−1^), and OH (3307 cm^−1^). There were few substantial differences between the V125 and V125E8 groups.

**Figure 5:**
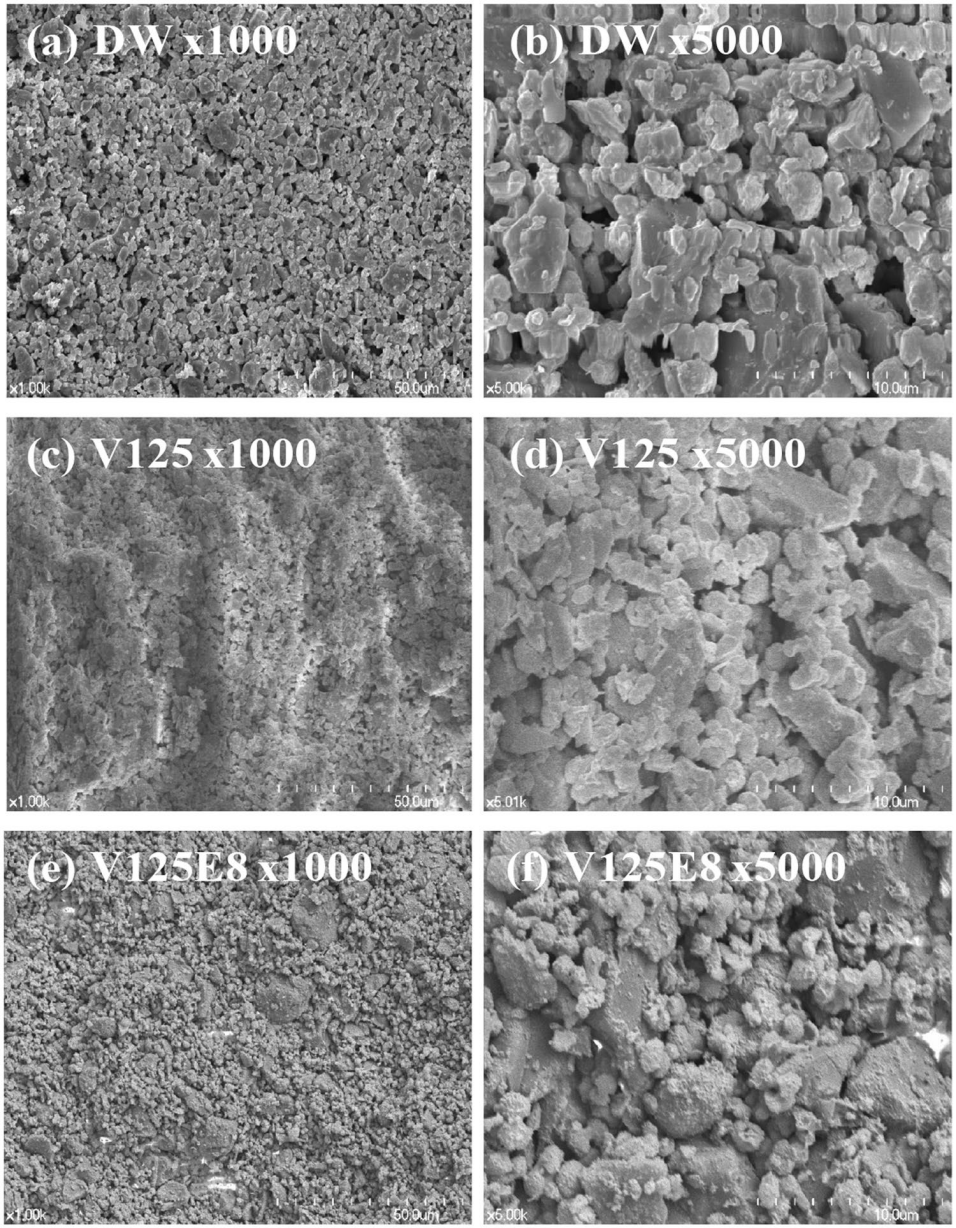
SEM images of the surface morphology of CPC composites. Larger pores 2–3 μm in diameter are visible in the DW samples (**a**,**b**). The V125 and V125E8 samples seem denser compared to the DW samples, as they appear to have fewer pores (**c**–**f**). V125E8 appeared to have larger crystallites with less sharp edges than DW or V125, and to have a slightly denser surface than V125.

Figure 5 shows the microstructures of the fractured surfaces of the three experimental groups. Larger pores 2 ~ 3 μm in diameter were visible in the DW group (Fig. 5a,b). The V125 and V125E8 groups seemed much denser compared to the DW group, as they appeared to have fewer pores (Fig. 5c–f). V125E8 appeared to have larger crystallites with fewer sharp edges than DW and V125 (Fig. 5f), and to have a slightly denser surface than V125. This surface appearance may have resulted from the likely ability of the ELP chains to stabilize DCPA and TTCP, which results in increased crystallite formation.

### Biocompatibility of ELP/CPC Composites

**Figure 6:**
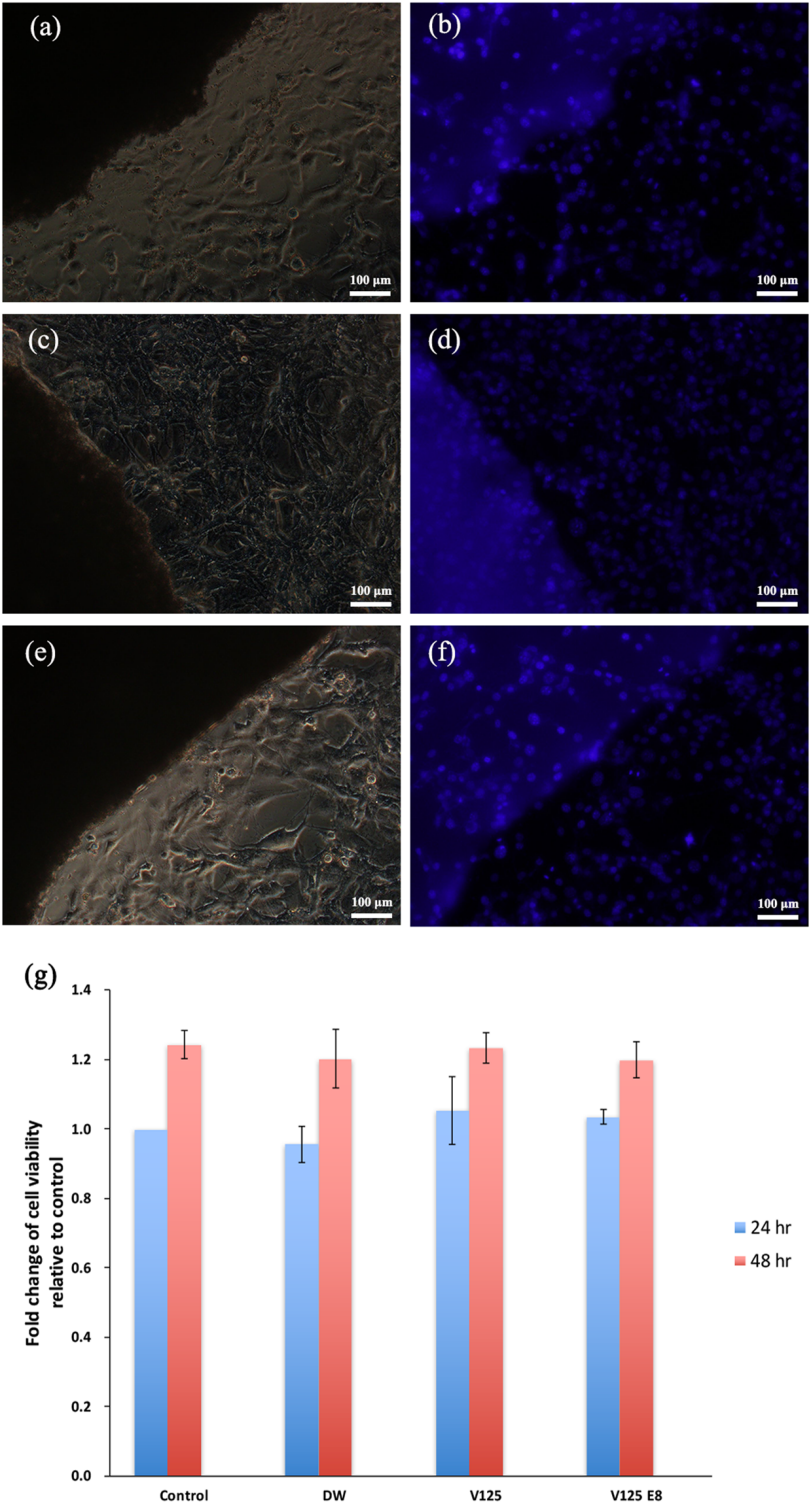
Cell attachment and viability tests. Parts (**a**–**f**) show attached cells at the CPC boundary in light microscope and fluorescence images of the same area [DW group, (**a**) and (**b**); V125, (**c**) and (**d**); V125-E8, (**e**) and (**f**)]. NIH3T3 cells showed good distribution on CPC; no specific differences among groups were found. The cell viability increased after 48 h compared to 24 h in all groups (*p* < 0.05), and there was no significant difference among groups (*p* > 0.05) (**g**).

Fluorescence staining was used to observe the morphology of cells cultured on CPC mixed with the 3 types of liquid. As shown in Fig. 6, the cells remained viable; a normal morphology and spreading pattern were observed on the CPC, regardless of the ELP supplementation used. No nuclear condensation changes were observed in cells grown on ELP-supplemented CPCs. There was no difference in cell distribution on the composites compared to control cells grown on the plastic culture substrate.

The viability of cells cultured for 24 and 48 h on control and supplemented CPCs was quantitatively assessed by measuring the mitochondrial dehydrogenase activity with the WST-1 assay. There was no significant difference among the behaviours of the three groups. The viability of the cells grown on all groups of CPC significantly increased after 48 h compared to viability at 24 h (*p* < 0.05) and there was no difference in viability at 48 h among groups (*p* > 0.05) (Fig. 6g).

## Discussion

The medical or dental use of CPC can be bolstered by improving its physical properties. Although there have been numerous attempts to improve the mechanical characteristics of CPCs by incorporating specific additives, the resulting composites leave much to be desired^3,5,9,10^. In this study, we demonstrated that supplementation of CPC with ELPs significantly improved the mechanical properties of the CPC. Specifically, the incorporation of V125E8 increased the microhardness by approximately 2- to 7-fold and the compressive strength by 10-fold, which is of clinical importance considering that compaction pressure was not applied in our experimental setting. The compaction force usually reduces the porosity of CPC and increases the mechanical properties^22^. Due to the properties of our ELP/CPC, it can be applied as thin layers in small areas such as tooth defects without the need for compaction force, which is regarded as clinically beneficial.

Generally, the strength of CPC increases due to a decrease in porosity, which was also observed in this study. The V125E8-supplemented CPC, which had the highest strength and hardness, had fewer pores and a more compact surface structure compared to the V125 and DW groups. ELP may be physically incorporated into the crystal lattice as the crystals precipitate^23^. In the case of V125E8, the interaction of the carboxylic acid residues in the octaglutamic acid oligopeptide (E8) and the calcium ions released from the CPC may reinforce the inter-crystal binding, which might have resulted in increased strength and hardness^24^. Previous studies reported that CPC embedded in a polymeric matrix has greater physical properties^25–27^. There is also a possibility that the ELP functioned as a polymeric matrix to firmly hold growing HA crystals^28^. Meanwhile, V125E8 appears to have acted as a plasticizer. When V125E8 was used as a liquid, more powder could be incorporated and an L/P ratio of less than 0.3 was possible; this ratio was not achieved for V125 or DW. V125E8 also created a more viscous CPC compared to other groups when mixed at the same L/P ratio, although this study did not quantitatively compare the rheological properties. Plasticizers are known to expose a greater surface area for hydration of CPC by dispersion action, resulting in improved mechanical strength^7,29,30^. The negatively charged octaglutamic acid peptides with high calcium affinity in V125E8 seem to act as this intrinsic plasticizer, resulting in the increases in strength and hardness observed in this study.

An increase in the L/P ratio tends to result in decreased mechanical properties in correlation with the higher porosity of the microstructure^30,31^. This trend was broadly observed in our study; the exception was specimens with an L/P ratio of 0.4, which showed a higher microhardness and compressive strength than those with a ratio of 0.3. We hypothesize that powder in excess of the optimal L/P ratio of 0.4 might interfere with complete hydration of the CPC, which can result in decreases in hardness and strength. Therefore, the suggested L/P ratio of CPC for optimal mechanical properties would be around 0.4, regardless of ELP supplementation.

CPCs are bioactive cements implanted as they are setting; they fully set in tissue fluid, blood, and low pH conditions. Thus, the washout resistance and stability of the cement in physiological environments are important requirements for clinical use^32^. Our results indicate that the incorporation of ELPs improves the washout resistance of CPCs; in particular, the V125E8 group was at least 3-fold more stable than the V125 group. We assume that the same mechanism that results in improved mechanical properties also enhances washout resistance. The incorporation of ELPs into the crystal lattice, or the chelation reaction of carboxylates in the glutamic acid residues of V125E8, and calcium ions might also improve the viscoelasticity and impede the penetration of CPC paste by liquids during the setting process. In addition, the SEM images of V125- and V125E8-supplemented CPC composites revealed a denser microstructure with a smaller pore size and dispersed deposits of a shaggy surface compared to the DW controls. It suggests that V125 and V125E8 altered the composition of the CPC. FT-IR analysis revealed the chemical compositional changes due to ELP supplementation; the V125 and V125E8 groups commonly presented additional peaks at 1656 cm^−1^ and 1525 cm^−1^ from amide absorption^33,34^. Similar peaks were not observed in the DW group. Another possible explanation for improved washout resistance in ELP-supplemented CPC is related to the transition temperature of the ELP. The transition temperature of V125E8 was determined to be 33 °C in a previous study^21^. Soaking in 37 °C HEPES solution would promote peptide transition to an aggregated hydrophobic state, resulting in washout resistance. This transition of ELPs below the physiologic temperature can be highly beneficial if the CPC is mixed at a low temperature and applied in surgical sites.

In this study, supplementation with ELPs increased the setting time of the CPC, and V125E8 presented a more pronounced delay in setting time compare to V125. It can be reasoned that the interaction between the ELP and HA formed an inorganic matrix that not only improved the mechanical properties, but also decreased the hydration reaction of the CPCs. The diffusion of calcium and phosphate ions in the CPC is hindered due to the increased viscosity, which may increase the duration of the gel phase of the cements, which is the stage of incomplete setting^32^. As mentioned previously, E8 contributes to more viscoelastic characteristics of ELP/CPC composites due to its high affinity to calcium ions. This characteristic would improve the material’s washout resistance, but would also further increase the setting time. The prolonged setting time of our ELP/CPC composites could be a challenge for its clinical applications. Before setting, the ELP/CPC might have the possibility to be damaged even under light physical pressure due to incomplete setting of the composite. Lowering the L/P ratios could be a way to decrease the delaying effect of the ELP on the setting time. As the L/P ratio decreased, the CPCs had shorter setting times and the differences in setting times between the ELP-supplemented and control groups decreased. In the future, we can include a setting accelerator such as sodium phosphate dibasic (Na_2_HPO_4_) or calcium chloride (CaCl_2_) to overcome the limitation of setting time in clinical applications^35–37^. Further study of this newly synthesized composite is needed.

During or after ELP/CPC setting, ionic exchanges and fluctuations in pH might cause cellular cytotoxic effects^38^. Based on the examination of the biocompatibility of ELP/CPC composites by cell attachment analysis and the cell viability tests in this study, ELP-supplemented CPCs were non-cytotoxic and supported cell attachment. As biocompatibility is a primary advantageous characteristic of CPCs, an improvement in the mechanical property without any adverse effect on the biocompatibility of ELP/CPC composites might indicate substantial potential benefits in clinical applications, though additional complementary investigations are necessary.

In conclusion, the incorporation of the ELPs, V125 and V125E8 into CPCs enhances the mechanical properties of the CPC without hampering biocompatibility. We strongly believe that the enhanced mechanical properties and anti-washout behaviour of our ELP/CPC composites increases the clinical applicability of CPCs and warrants further *in vitro* and *in vivo* studies. Despite favourable improvements due to the addition of ELP, the limitation of the increased setting time, especially due to the addition of V125E8, must be overcome through further studies using hardening accelerators. Within the scope of this study, the best L/P ratio of ELP-supplemented CPC for clinical applicability is approximately 0.4, considering both the improvement in mechanical properties and the shorter setting time. We believe our results are applicable to the field of applied biomaterials science and will have diverse applications in biology and medicine.

## Methods

### ELP synthesis

The ELP backbone consists of repeating sequences of VPGXG; the guest amino acid X is a valine (V). The backbone is named using the single-letter code for the guest amino acid and the number of pentapeptides in the sequence. For example, V125 contains 125 repetitions of the sequence VPGVG. Modification of the protein terminal is indicated by the addition of the amino acid letter code and a number; V125E8 contains an E8 functional group at the C-terminal of the V125 sequence.

**Figure 7:**
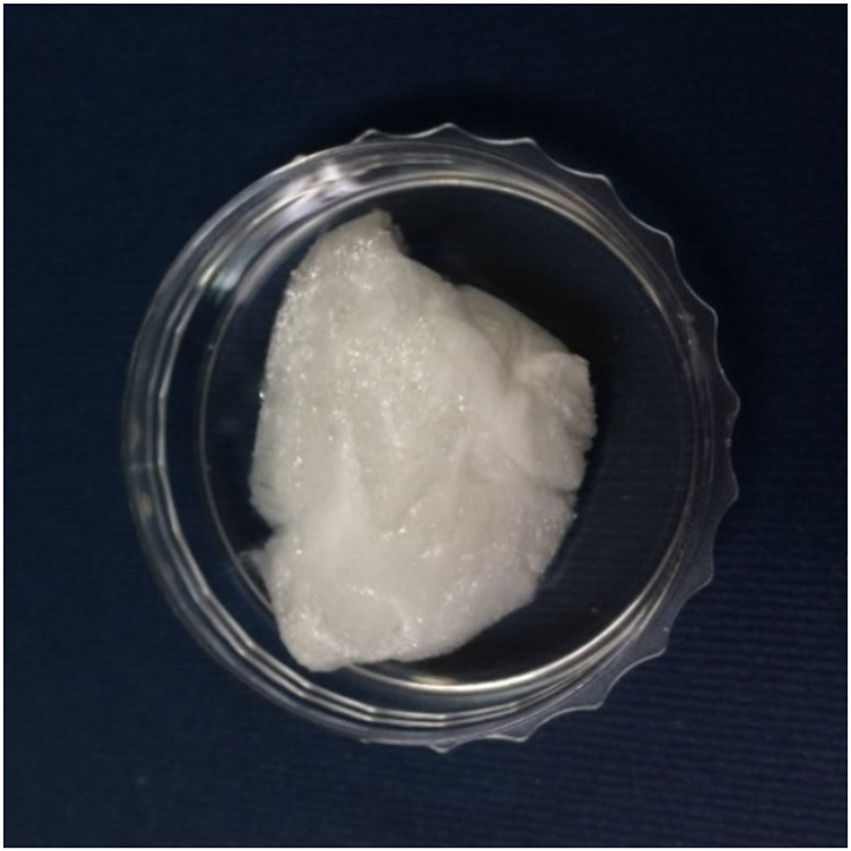
Lyophilized ELPs. The ELPs were synthesized, purified, and dialyzed with deionized water, followed by lyophilisation. A 10 wt% solution of lyophilized ELP was used as a liquid phase for the preparation of ELP-CPC composites.

The two types of ELPs, V125 and V125E8 were used in this study because they effectively formed ELP-CPC composites in our previous study^21^. The ELP synthesis procedure was performed according to the method described previously^21^. Briefly, DNA sequences corresponding to the sequences of V125 and V125E8 were created by annealing and ligation of synthetic oligonucleotides (IDT, Inc., Coralville, IA, USA). The DNA sequences encoding V125 and V125E8 were transferred to modified pET28b vectors (EMD Millipore, Gibbstown, NJ, USA) containing specific N-terminal and C-terminal DNA sequences. The ELP plasmids were transformed into BLR (DE3) *E. coli* (EMD) for expression. The *E. coli* were cultured in Terrific Broth at 37 °C for 24 h^39^. The ELPs were then purified as described previously^21^. Briefly, the *E. coli* were isolated by centrifugation, resuspended in 10 mM Tris-Cl, 2 mM EDTA, pH 8.0 and lysed by probe sonication for 3 × 30 sec with a 2 min interval between each sonication (Sonicator 3000, Misonix, Inc., Farmingdale, NY, USA). Cell debris was removed by centrifugation, and DNA was precipitated using polyethylenimine (Sigma-Aldrich, Steinheim, Germany). The supernatant was further purified using 3 rounds of inverse transition cycling as described by Meyer and Chilkoti^40^. The proteins were dialyzed with deionized water and then lyophilized. The lyophilized ELPs and their amino acid sequences are shown in Fig. 7 and Table 1. As in the previous study, a 10% solution of ELP by weight in deionized water was used as the liquid phase for the ELP-CPC composites to achieve optimal handling and mechanical properties^21^.

**Table 1:** The amino acid sequences of synthesized ELPs.

Name	N-terminal	Backbone	C-terminal
V125	MSGVG	(VPGVG)_125_	VPG
V125E8	MSGVG	(VPGVG)_125_	VPGSEEEEEEEE

### ELP/CPC Composite Synthesis

**Figure 8:**
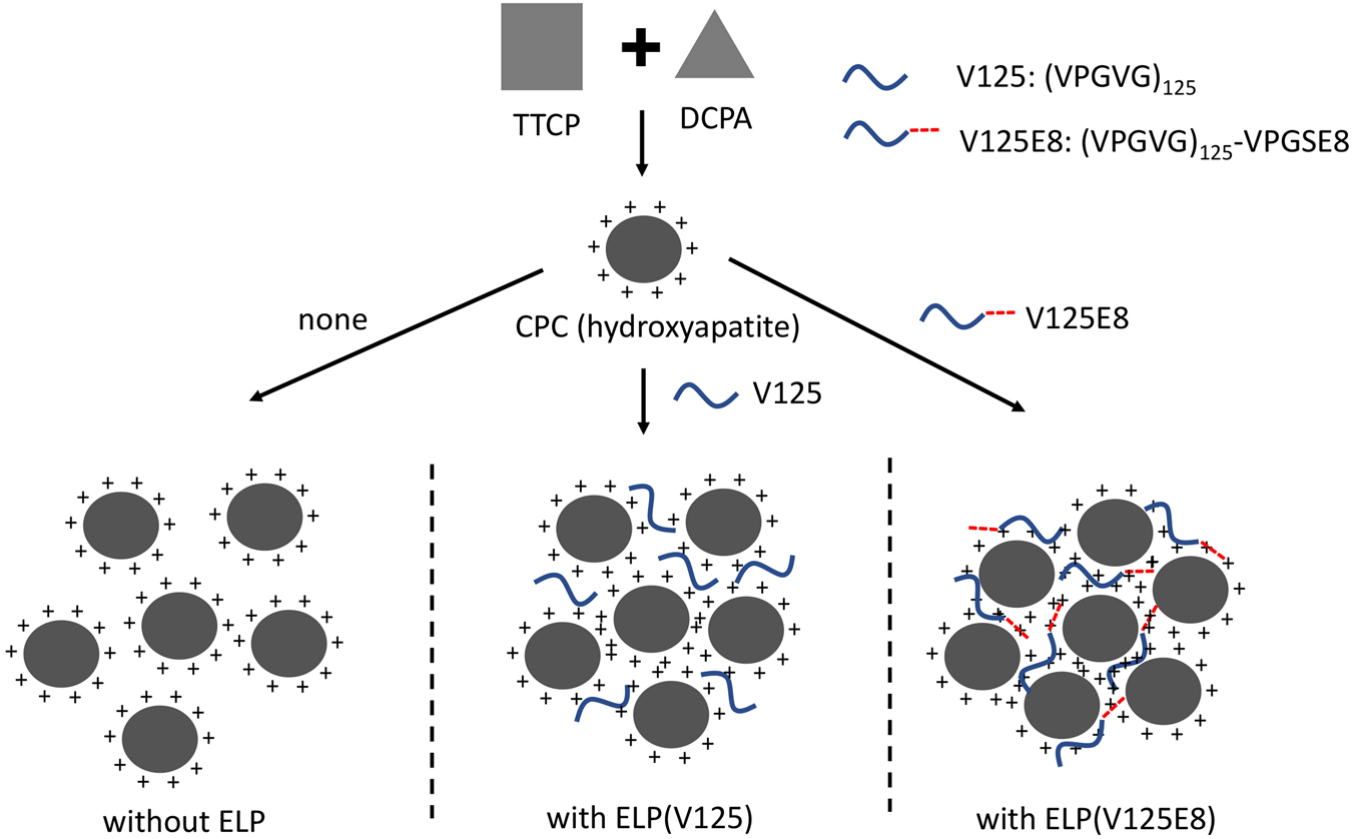
Schematic diagram of CPC supplemented with ELPs (V125 or V125E8). CPC containing a mixture of TTCP and DCPA sets as hydroxyapatite. ELP with negatively charged octaglutamic acid (V125E8) interacts with calcium ions of hydroxyapatite, which improves the solubility of CPC and creates a more compact structure.

The CPC powder was prepared by mixing TTCP and DCPA at a molar ratio of 1:1. TTCP (Hangzhou Union Biotechnology Co., Ltd., Hangzhou, China) with an average particle size of ~15 μm was used. DCPA was synthesized from a solution of CaCO_3_ and H_3_PO_4_; the reaction time was 24 h^41^. The powder was mixed with 3 types of liquid to produce three different cements. Accordingly, the samples were divided into three groups: the DW group (CPC mixed with deionized water), the V125 group (CPC mixed with 10% V125 solution by weight), and the V125E8 group (CPC mixed with 10% V125E8 solution by weight). The 10%-by-weight ELP solutions were used because increasing viscosity at greater concentrations makes the solution difficult to mix. The three experimental groups were subdivided according to L/P ratios of 0.3, 0.4, 0.5, 0.6, and 0.7. Consistent mixing was difficult at an L/P ratio < 0.3, and the mixture was too watery to handle at ratios > 0.7. The CPC supplemented with ELPs is theoretically depicted in Fig. 8.

### Physical Properties of ELP/CPC Composites

#### Microhardness

To examine the microhardness of the ELP/CPC composites, five specimens of each group were prepared. The mixtures were placed in plastic moulds 5.0 mm in diameter and 3.0 mm in height. The moulds were stored in 100% humidity chambers at 37 °C for 4 days. A load of 240 mN was applied to specimens for 10 seconds using the pyramidal diamond indenter of a microhardness tester (HMV-2, Shimadzu Scientific Instruments, Kyoto, Japan). The Vickers microhardness value (Hv) was calculated using the following formula:

$${\rm{Hv}}=0.1891\times \frac{{F}}{{{d}}^{2}}$$

where 0.1891 is the Vickers constant (from indenter geometry); *F* is the force in Newtons (=0.9807 N for the 100-g weight used); and *d* is the arithmetic mean of the two diagonals of the impression made by the indenter, in millimetres.

#### Compressive strength

To examine the compressive strength of the ELP/CPC composites, 10 samples were prepared for each group. The mixtures were placed in Teflon-coated plastic moulds 3.0 mm in diameter and 7.0 mm in height and stored for 4 days in 100% humidity chambers at 37 °C. The mixed CPCs were placed in the moulds carefully to prevent the application of pressure and avoid an increase in strength due to compaction of the cement. The samples were removed from the moulds and tested at a crosshead speed of 1 mm/min using a computer-controlled universal testing machine (AGS-X, Shimadzu Scientific Instruments). The maximum load required to fracture each sample was measured and the compressive strength was calculated in MPa.

#### Setting time measurements

To measure the setting time, the mixtures were placed in plastic moulds 5.0 mm in diameter and 3.0 mm in height. The setting time was measured using Gillmore needles (HJ-1120, Heungjin Co., Ltd., Gyeonggi-do, Korea) according to ASTM International C266-99^42^. A 113.4-g Gillmore light needle with a cylindrical tip, 2.12 mm in diameter, was applied vertically to the horizontal surface of the cement to measure the initial setting time; the needle initially marked the surface with an indentation. The final setting time was then measured with the same technique, using a 453.6-g Gillmore heavy needle with a cylindrical tip, 1.06 mm in diameter. The cement was considered to have set when this mark could not be seen on the specimens. A total of six specimens for each group was measured.

#### Washout resistance test

CPC was mixed with either DW, V125, or V125E8 at an L/P ratio of 0.5. Newly mixed CPC samples were shaped into small discs ~5 mm in diameter by dropping samples into a petri dish and tapping on the floor. A HEPES solution (Sigma-Aldrich) of 37 °C was poured into the petri dish and the CPC was observed at 5 min, 1 h, and 24 h. After 24 h, the intact parts of the CPC were removed, and the disintegrated parts were dried in an oven at 37 °C for 48 h. The dried cement remnants were weighed, and the percentage of washout of each cement was calculated. Each sample type was measured in triplicate.

#### Structural analysis

FT-IR spectroscopy was used to analyse the chemical characteristics of the composites (FT-IR spectrophotometer IF66, Bruker Optics, Billerica, MA, USA). Samples of the three composites with L/P ratios of 0.5 were stored in 100% humidity chambers at 37 °C for 4 days, and then dried in a 37 °C oven for 24 h. Each dried sample was crushed and combined with potassium bromide powder to make a pellet. The measurements were made in transmission mode between 400 and 4000 cm^−1^ with a resolution of 4 cm^−1^; 64 scans were performed.

The surface morphology of the ELP/CPC composites was observed with SEM (S-4700, Hitachi, Tokyo, Japan). Hardened samples at an L/P ratio of 0.5 were fractured horizontally to expose the interior surface for observation. The samples were sputter-coated with gold before SEM observation.

### Biocompatibility of ELP/CPC Composites

The cell morphology of NIH3T3 cells cultured on the surfaces of CPC specimens of the three experimental groups (L/P ratio = 0.5) was observed. After incubation for 24 h, the cells were washed with PBS, fixed in 4% paraformaldehyde (Sigma-Aldrich Corp., St. Louis, MO, USA) for 5 min, and then permeabilized with PBS containing 0.1% Triton X-100 (Sigma-Aldrich Corp.) for 20 min. Nuclei were stained with 4′,6-diamidino-2-phenylindole (DAPI; Invitrogen Corp., Carlsbad, CA, A). The morphology was observed using a confocal laser fluorescence microscope (D-Eclipse C1, Nikon Instruments, Inc., Tokyo, Japan).

Quantitative cell viability was determined using a WST-1 assay (reagent grade, Dojindo Molecular Technologies, Inc., Gaithersburg, MD, USA). NIH3T3 cells were seeded onto the three experimental specimens, which were prepared at an L/P ratio of 0.5, at a density of 3 × 104 cells/mL in 96-well plates and incubated in DMEM at 37 °C and 100% humidity with 5% CO_2_ for 24 and 48 h. After incubation, 0.1 mL of WST-1 solution was added, and the specimens were incubated for 2 h. Absorbance was measured at 450 nm.

### Statistical analysis

All measured values of the microhardness test, compressive strength test, washout resistance test, setting time, and cell viability tests were statistically analysed with a one-way and/or two-way ANOVA using SPSS IBM Statistics for Windows, Version 22.0 (IBM Corp., Armonk, NY, USA). Post-hoc comparisons were performed using Tukey’s HSD test. A *p-*value < 0.05 was considered significant.
